# Supplementary material for: Very Large Pore Mesoporous Bioactive Silicate Glasses: Comparison of Behavior toward Classical Mesoporous Bioactive Glasses in Terms of Drug Loading/Release and Bioactivity
Source: Materials (Basel). 2024 Jan 11;17(2):373. doi: 10.3390/ma17020373 (PMC10820009; doi:10.3390/ma17020373)
Supplement: Supplementary file 1 [file materials-17-00373-s001.zip › materials-2772691-supplementary.pdf]

SUPPLEMENTARY MATERIALS

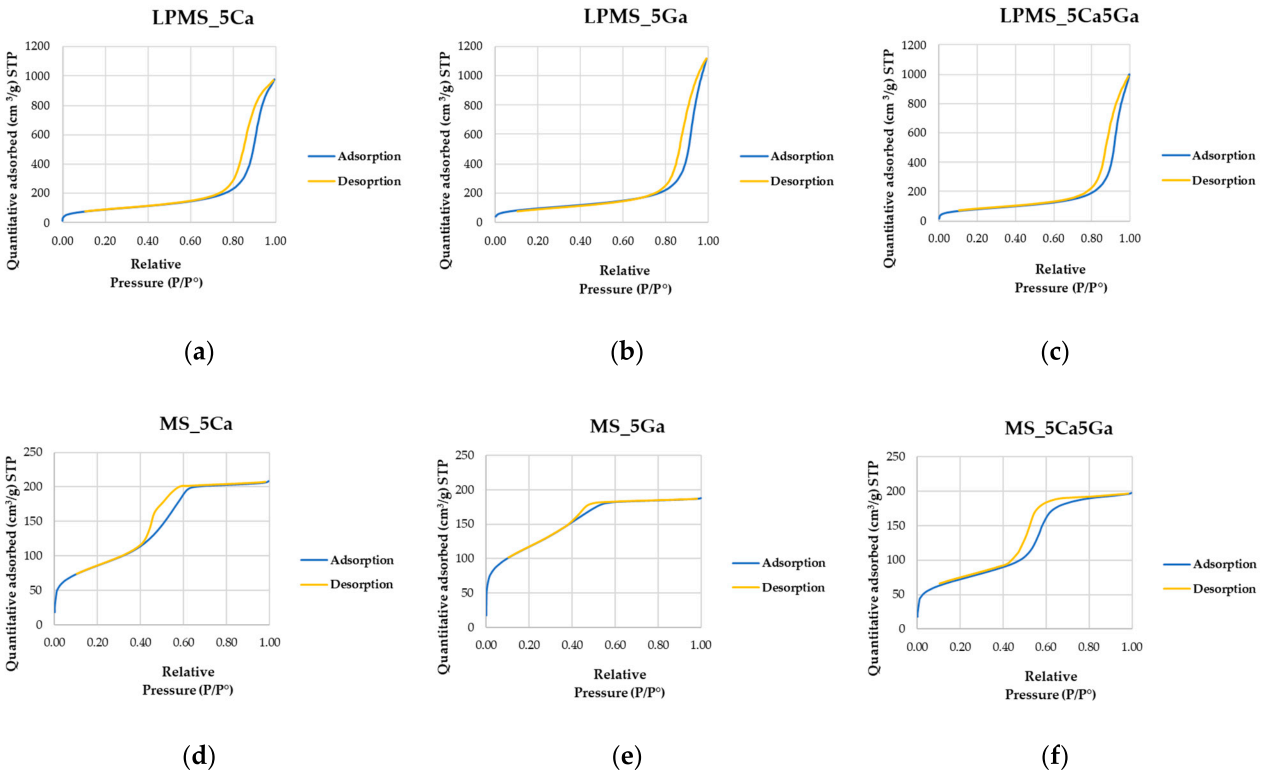

Figure S1. BET surface area analysis results: N_2-adsorption–desorption isotherm plots of samples (**a**) LPMS_5ca, (**b**) LPMS_5Ga, (**c**) LPMS_5Ca5Ga, (**d**) MS_5Ca, (**e**) MS_5Ga, (**f**) MS_5Ca5Ga.

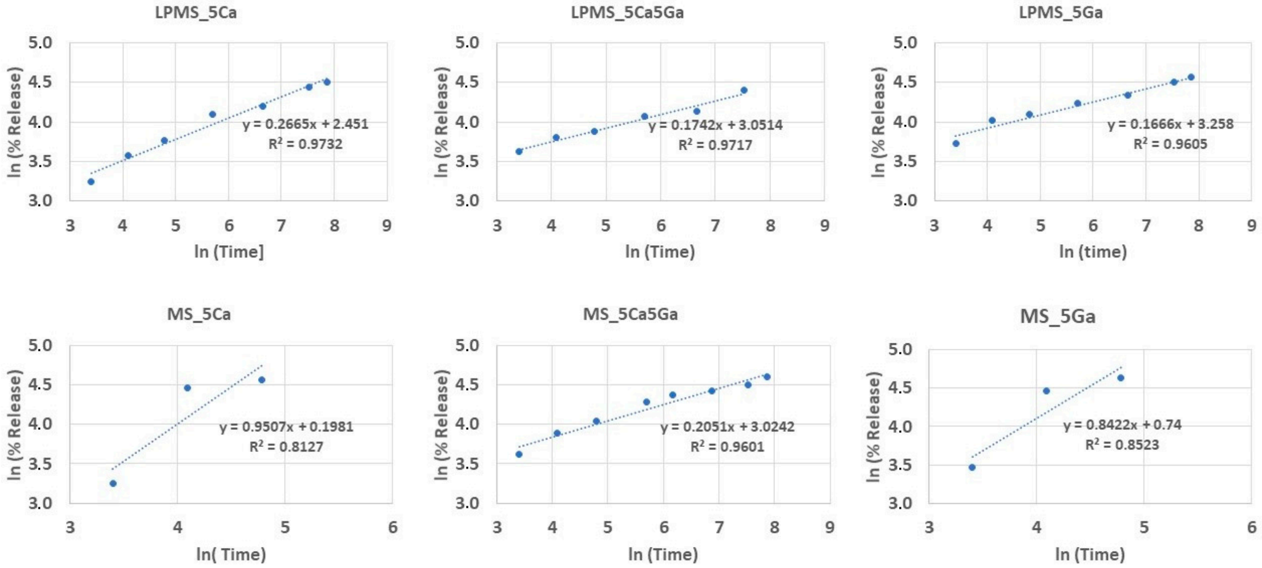

Figure S2. Korsmeyer-Peppas model release kinetics of nisin aspirin obtained for LPMS and MS samples.

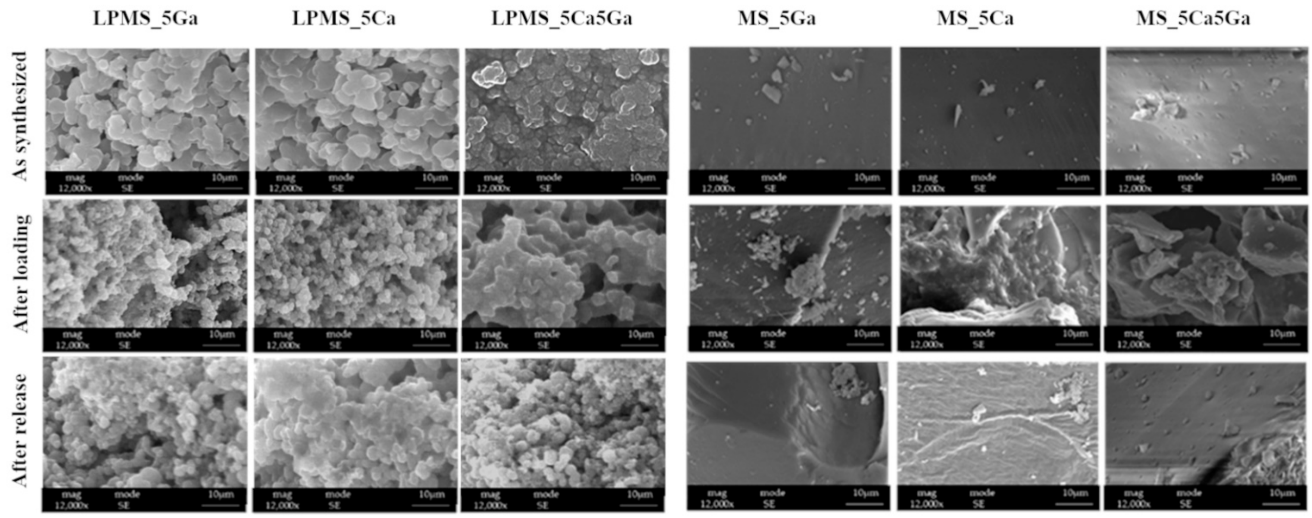

(**a**) (**b**) (**c**) (**d**) (**e**) (**f**)

Figure S3. SEM-FEG images of (**a**) LPMS_5Ga, (**b**) LPMS_5Ca, (**c**) LPMS_5Ca5Ga, (**d**) MS_5Ga, (**e**) MS_5Ca, (**f**) MS_5Ca5Ga. In the first row are reported images of as synthesized structures, in the second are reported loaded structures, and in the last row are reported structures after nisin release.

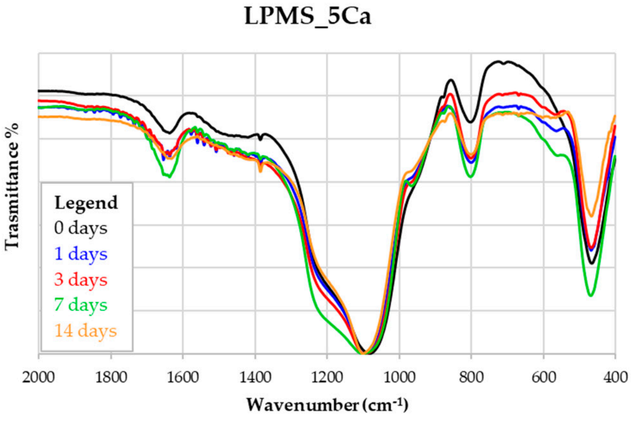

(**a**)

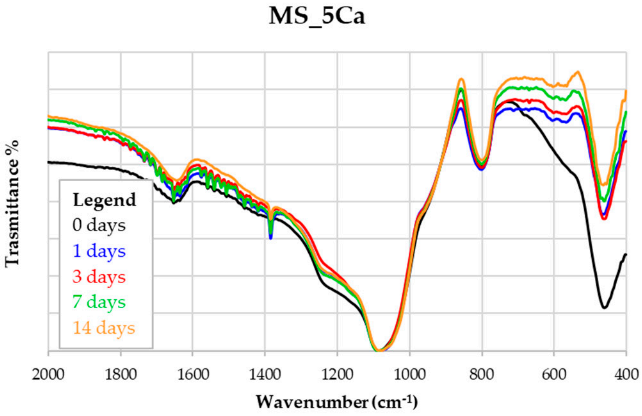

(**b**)

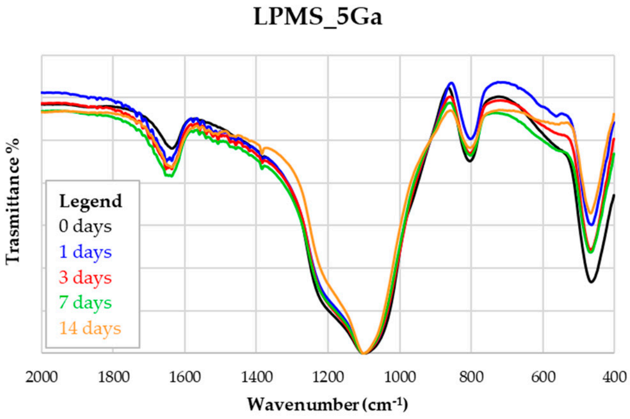

(**c**)

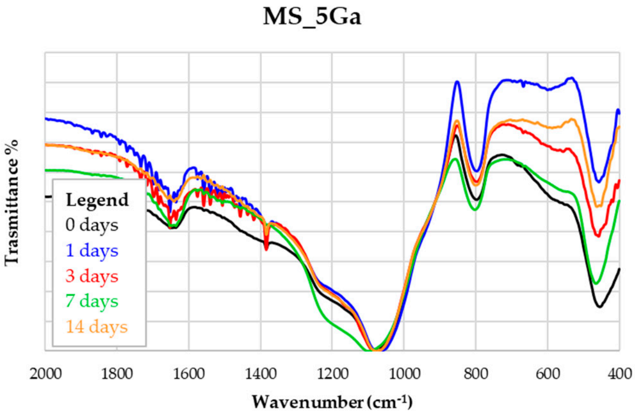

(**d**)

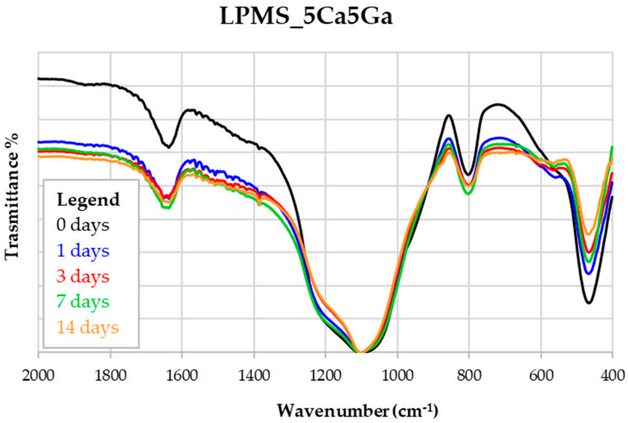

(**e**)

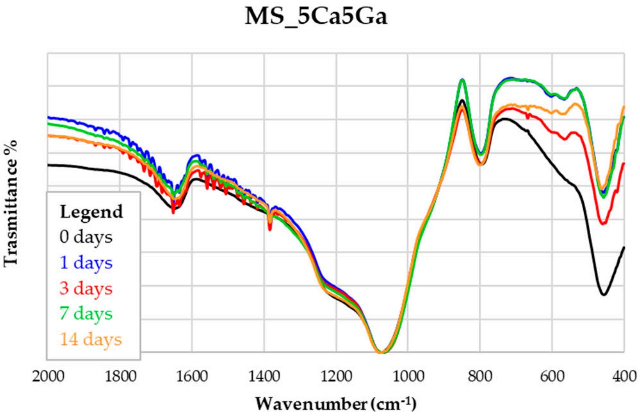

(**f**)

Figure S4. FI-IR spectra of all samples were acquired at 1, 3, 7, and 14 days for samples prepared as KBr pads. In order: (**a**) LPMS_5Ca, (**b**) MS_5Ca, (**c**) LPMS_5Ga, (**d**) MS_5Ga, (**e**) LPMS_5Ca5Ga and (**f**) MS_5Ca5Ga.

Table S1. SSA_{BET} and Total Pores Area determined respectively with N_2 adsorption/desorption and Hg-Porosimetry.

Sample	SSA_{BET} N_2 ads./des. (m^2/g)	Pore Size N_2 ads./des. (nm)	Pore Volume N_2 ads./des. (cm^3/g)	Total Pores Area Hg-Porosimentry (m^2/g)
**LPMS_5Ca**	323	16.3	1.56	392
**LPMS_5Ga**	324	19.2	1.77	376
**LPMS_5Ca5Ga**	283	19.1	1.56	344
**MS_5Ca**	306	4.0	0.31	73
**MS_5Ga**	417	3.0	0.23	53
**MS_5Ca5Ga**	252	4.8	0.29	179
